# Patient preferences for the allocation of deceased donor kidneys for transplantation: a mixed methods study

**DOI:** 10.1186/1471-2369-13-18

**Published:** 2012-04-18

**Authors:** Allison Tong, Stephen Jan, Germaine Wong, Jonathan C Craig, Michelle Irving, Steve Chadban, Alan Cass, Niamh Marren, Kirsten Howard

**Affiliations:** 1 Sydney School of Public Health, The University of Sydney, Sydney, NSW 2006, Australia; 2 Centre for Kidney Research, The Children's Hospital at Westmead, Sydney, NSW 2145, Australia; 3 Renal and Metabolic Division, The George Institute for Global Health, Sydney, NSW 2050, Australia; 4 Centre for Transplant and Renal Research, Westmead Hospital, Sydney, NSW 2145, Australia; 5 Central Clinical School, Bosch Institute, The University of Sydney, Sydney, NSW 2006, Australia

## Abstract

**Background:**

Deceased donor kidneys are a scarce health resource, yet patient preferences for organ allocation are largely unknown. The aim of this study was to determine patient preferences for how kidneys should be allocated for transplantation.

**Methods:**

Patients on dialysis and kidney transplant recipients were purposively selected from two centres in Australia to participate in nominal/focus groups in March 2011. Participants identified and ranked criteria they considered important for deceased donor kidney allocation. Transcripts were thematically analysed to identify reasons for their rankings.

**Results:**

From six groups involving 37 participants, 23 criteria emerged. Most agreed that matching, wait-list time, medical urgency, likelihood of surviving surgery, age, comorbidities, duration of illness, quality of life, number of organs needed and impact on the recipient's life circumstances were important considerations. Underpinning their rankings were four main themes: enhancing life, medical priority, recipient valuation, and deservingness. These were predominantly expressed as achieving equity for all patients, or priority for specific sub-groups of potential recipients regarded as more "deserving".

**Conclusions:**

Patients believed any wait-listed individual who would gain life expectancy and quality of life compared with dialysis should have access to transplantation. Equity of access to transplantation for all patients and justice for those who would look after their transplant were considered important. A utilitarian rationale based on maximizing health gains from the allocation of a scarce resource to avoid "wastage," were rarely expressed. Organ allocation organisations need to seek input from patients who can articulate preferences for allocation and advocate for equity and justice in organ allocation.

## Background

Kidney transplantation improves survival and quality of life for patients with end-stage kidney disease [1]. Deceased donor kidneys are a scarce health resource, yet patient preferences for organ allocation are largely unknown [2,3]. Therefore, a potential mismatch in the underlying issues that shape preferences regarding organ allocation compared with healthcare professionals and policy makers may be overlooked.

Patient involvement in healthcare and policy is widely advocated to improve patient satisfaction and relationship with healthcare professionals [4,5]. As "end-users," patients with end-stage kidney disease are a key stakeholder group; therefore eliciting their perspectives in deceased donor kidney allocation is ethically warranted. Deceased donor organ allocation algorithms are developed with little direct input of patient values and are based on a combination of the following criteria: waiting time, medical urgency, human leukocyte antigen (HLA) matching, sensitization and paediatric status [6]. This lack of patient input into transplantation policy, combined with a lack of evidence around the nature of patient preferences, has added potentially unnecessary controversy to the formulation of policy in this area [7-10]. Recently, the Kidney Committee of the Organ Procurement and Transplantation Network in the United States proposed an allocation system that would "preferentially allocate the top quintile of donor kidneys with the best expected graft survival to the top quintile of transplant candidates with the longest predicted post-transplantation survival. For the remaining donors and recipients, there would be broad age-matching within a 30-year age range" [11]. This seeks to maximize graft survival but it limits the overall transplant opportunities for older and sicker patients, and may not lead to maximising incremental health gains.

In Australia, deceased donor kidney allocation is coordinated through the National Organ Matching System. In the first instance, the National Kidney Interstate Exchange program identified suitable kidney for patients who have an extremely high level of HLA-antibodies. However, approximately 80% of kidneys are allocated within the state in which they are donated. All states ensure that their algorithm results in a minimum of 30% of patients receiving kidneys on the basis of waiting time [12].

The extent to which such current allocation protocols and debates actually reflect patient values remains uncertain. The few studies which have evaluated patient perspectives on organ allocation have found that patients favour a balance of antigen matching and waiting time to achieve fairness for potential recipients [13] and a vast majority disagree with use of recipient age as a criteria, and an over-weighting on HLA matching compared with time on the waiting list [3].

This study aims to determine patient preferences for the allocation of deceased donor kidneys for transplantation and the reasons for their rankings. This may facilitate current allocation protocols to be made consistent with patient preferences and inform the development and implementation of strategies that are thus more explicitly cognizant of patient values.

## Methods

This study used a mixed methods approach using combined focus/nominal group technique. Pairing quantitative and qualitative techniques is useful for generating more complete data by using results from each to mutually complement and corroborate findings [14,15].

### Participants

Participants were eligible if they were receiving haemodialysis, peritoneal dialysis or had received a kidney transplant; were English-speaking, and aged 18-80 years. They were recruited from two large transplanting centres (Westmead Hospital and Royal Prince Alfred Hospital) in New South Wales, Australia and purposively selected to achieve a range of age, sex, dialysis modality, and waitlist status. The participants were grouped according to treatment (dialysis or transplant). Participants were offered reimbursement for their travel expenses. Approval was obtained from The University of Sydney, Human Research Ethics Committee. All participants were asked to provide written informed consent prior to participation.

### Data collection

Each two-hour combined focus/nominal discussion had three phases, preliminary questions about perspectives on organ donation and allocation, identification of organ allocation criteria, and an individual ranking exercise of criteria identified from group discussion, using a modified nominal group technique. Nominal group techniques have been successfully applied to various areas of health research, and have specifically been used in prioritizing patient preferences for healthcare [15-17]. It has been recommended that a minimum of three groups of approximately 6-10 participants be convened for each participant group [18]. The question schedule was developed based on relevant literature [2,3,19,20] and discussion among the research team. The participants discussed attributes they believed were important for kidney allocation to generate a list which was augmented with attributes identified from the literature. Each participant individually ranked the attributes for the allocation of deceased organs in order of perceived importance. The feasibility of this ranking method has been demonstrated in previous nominal group technique studies [15]. All groups were convened in a hotel meeting room and facilitated by AT. An observer (MI, KH) recorded field notes on group dynamics and interactions, participant characteristics, body language and the context surrounding the discussion. All sessions were audio-recorded and transcribed verbatim. We convened nominal groups until data saturation, defined as the point in data collection when no new data were generated to bring additional insight to the research question.

### Analysis

Nominal group ranking: Individual participant rankings were used to calculate importance scores for each factor. The highest ranked factor for each respondent was given 15 points, the next most important given 14, and so on, progressively down to least important. If a factor was not mentioned, it was assigned an importance score of zero for all respondents in that group. Mean importance scores were calculated. The percentage of respondents who ranked a factor in their top 10 was also calculated. Differences across groups (current treatment modality (transplant or dialysis), age group (less than 50 years, 50+ years), and non-English speaking background), were assessed using analysis of variance (ANOVA) for differences in mean importance scores of factors and *χ*2 tests for differences in the proportions of respondents reporting factors in their top 10 rankings.

Qualitative analysis: Transcripts were entered into HyperRESEARCH (ResearchWare Inc. United States. Version 2.8.3) Transcripts were reviewed by AT/NM who searched for concepts, themes and ideas, and developed a preliminary coding scheme using an adapted grounded theory approach [21]. The preliminary coding was discussed among AT, NM, KH, and MI. AT/NM refined the coding structure until it captured all relevant concepts. Through a process of constant comparisons between individuals, groups and patient populations (dialysis patients, kidney transplant recipients), we inductively developed descriptive and analytical themes to identify the participant reasons underpinning their allocation attribute rankings.

## Results

The six focus groups involved 37 participants, aged from 25 to 71 years (mean 52.0 years); 20 (54.1%) were men. (Table 1) The focus composition is provided in Table 2. Of the 37 participants, 25 (68%) had been on or were currently on the transplant waiting list. Five participants brought their carer. Kidney transplant recipients represented 24 (64.9%) of participants and the attendance rate was 61.7%. Reasons for non-attendance included work commitments, illness, unable to arrange transport, and hospitalisation.

**Table 1:** Participant characteristics

Characteristics	Transplant Patients n = 24 (%)	Dialysis Patients n = 13 (%)	Total n = 37 (%)
**Sex**			

Male	12 (32.4)	8 (21.3)	20 (54.1)

Female	12 (32.4)	5 (13.6)	17 (45.9)

**Age**			

20-29	2 (5.4)	0 (0.0)	2 (5.4)

30-39	3 (8.1)	0 (0.0)	3 (8.1)

40-49	8 (21.6)	2 (5.4)	10 (27.0)

50-59	5 (13.6)	7 (18.9)	12 (32.4)

60-69	5 (13.6)	3 (8.1)	8 (21.6)

70-79	1 (2.7)	1 (2.7)	2 (5.4)

**Dialysis modality**			

Haemodialysis	13 (35.1)	8 (21.3)	21 (56.8)

Peritoneal dialysis	11 (29.7)	5 (13.6)	16 (43.2)

**Time on Dialysis (yr)**			

< 1	N/A	0 (0.0)	0 (0.0)

1 - 2	N/A	7 (18.9)	7 (18.9)

3 - 4	N/A	2 (5.4)	2 (5.4)

≥ 5	N/A	4 (10.8)	4 (10.8)

**Time on waiting list (y)**			

NA	5 (13.5)	2 (5.4)	7 (19.0)

< 1	4 (10.8)	0 (0.0)	4 (10.8)

1 - 2	6 (16.2)	8 (21.3)	14 (37.8)

3 - 4	4 (10.8)	1 (2.7)	5 (13.5)

≥ 5	5 (13.5)	2 (5.4)	7 (19.0)

**No. of previous transplants**			

0	23 (62.2)	11 (29.7)	34 (91.9)

1	1 (2.7)	2 (5.4)	3 (8.1)

**Donor**			

Live	10 (27.0)	N/A	10 (27.2)

Deceased	14 (37.8)	N/A	14 (37.8)

**Organs needed**			

Kidney only	22 (59.5)	13 (35.1)	35 (94.6)

Kidney and pancreas	2 (5.4)	0 (0.0)	2 (5.4)

**Table 2:** Focus group composition

Characteristics	Focus group 1	Focus group 2	Focus group 3	Focus group 4	Focus group 5	Focus group 6
**Total number of participants**	8	7	9	6	5	2

**Sex**						

Male	3	4	5	3	4	1

Female	5	3	4	3	1	1

**Age**						

20-29	0	0	2	0	0	0

30-39	1	1	1	0	0	0

40-49	4	3	1	1	1	0

50-59	2	2	1	4	3	0

60-69	1	1	3	1	1	1

70-79	0	0	1	0	0	1

**Renal replacement therapy (current)**						

Haemodialysis	0	0	0	3	4	1

Peritoneal dialysis	0	0	0	3	1	1

Kidney transplantation	8	7	9	0	0	0

### Nominal group ranking

A total of twenty three separate factors emerged from the group discussions (Table 3). The ranking of the factors does not indicate the direction of whether respondents thought a higher or lower priority should be given to recipients based on that factor, but rather indicates that respondents thought it was important that the factor was considered in allocation.

**Table 3:** Individual ranking of all attributes important to patients and caregivers regarding organ allocation

Rank	Allocation attributes	Mean priority score				% times ranked in top 10			
		**All**	**Post-transplant**	**Waiting list**	**p value**	**All**	**Post-transplant**	**Waiting list**	**p value**

1	Matching/blood type/tissue compatibility	13.97	14.74	12.62	0.032	94%	98%	84%	NS

2	Time on waiting list	11.08	10.91	11.38	NS	94%	73%	76%	NS

3	Need/Medical urgency	11.08	11.83	9.77	NS	89%	79%	65%	NS

4	Likelihood of surviving surgery	9.25	9.74	8.38	NS	78%	65%	56%	NS

5	Age/Life stage*	8.19	7.70	9.08	NS	67%	51%	61%	NS

6	Other illnesses, current health and fitness	7.50	8.04	6.54	NS	64%	54%	44%	NS

7	Duration of illness	7.03	6.74	7.54	NS	58%	45%	50%	NS

8	Quality of life	6.92	6.91	6.92	NS	53%	46%	46%	NS

9	Number of organs needed	6.14	8.45	2.23	< 0.0001	49%	56%	15%	0.001

10	Impact on recipients family/family circumstances/dependents	5.75	5.87	5.54	NS	50%	39%	37%	NS

11	Years of life after transplant - life expectancy	5.50	5.83	4.92	NS	36%	39%	33%	NS

12	Lifestyle factors (smoking drugs drinking obesity compliance)	4.81	4.04	6.15	NS	31%	27%	41%	NS

13	Post- transplant: Support, follow-up, adherence, ability to cope	4.33	4.35	4.31	NS	31%	29%	29%	NS

14	Whether first or subsequent transplants	2.25	1.00	4.46	0.002	17%	7%	30%	NS

15	Donor vs. non donor status	2.19	2.78	1.15	NS	14%	19%	8%	NS

16	Risk of losing graft/rejection/surgical risk	2.00	2.26	1.54	NS	17%	15%	10%	NS

17	Contribution to community	1.89	1.30	2.92	NS	6%	9%	19%	NS

18	Distance/geography/logistics: get to hospital on time	1.36	1.13	1.77	NS	6%	8%	12%	NS

19	Psychological state of recipient/major psychological illness	1.22	1.91	0.00	0.029	8%	13%	0%	NS

20	Likelihood of recurrent disease in new organ/cause of disease	1.19	1.87	0.00	0.038	6%	12%	0%	NS

21	Religion/race	0.53	0.70	0.23	NS	0%	5%	2%	NS

22	Sex	0.44	0.52	0.31	NS	0%	3%	2%	NS

23	Weight	0.00	0.00	0.00	NS	0%	0%	0%	NS

*could be preference given to younger & children/or some say older because they can't afford to wait too long; NS, not statistically significant

#### Mean importance score

Respondents believed the most important factor to be considered in allocation was the extent of matching, or compatibility with a mean importance score of 14.0 from a maximum of 15.0. Other factors that respondents believed should be considered in allocation were length of time on the waiting list, the need/medical urgency of the patient and the age of the recipient. The current treatment modality (existing transplant or waiting list) significantly influenced the mean importance score for some factors (Table 2). For example, those with an existing transplant ranked the importance of matching/compatibility (p = 0.032) and the number of organs required (p < 0.0001) higher than those on the waiting list; but ranked the importance of the distinction between first and subsequent transplants significantly lower than those on the waiting list (p = 0.002). Respondents aged less than 50 ranked the importance of considering age of recipient in allocation significantly lower (mean rank 6.36) than respondents aged 50 or older (mean rank 9.36) (p = 0.036). Age of respondents and non-English speaking background did not significantly influence the mean importance score of any of the other factors identified.

#### Proportion of respondents ranking factors in top 10 most important factors for influencing the allocation decision

The proportion of respondents ranking a factor in their top 10 is also reported in Table 2. The proportion of respondents with an existing transplant who ranked the number of organs needed in their top 10 was significantly higher than for those on the waiting list (*χ*^2 ^(1df) = 11.42, p = 0.001) (Table 2); the proportion of respondents aged 50+ years who reported age of recipient in their top 10 was significantly higher than for those aged less than 50 years (*χ*^2 ^(1df) = 5.64, p = 0.026). Non-English speaking background did not significantly influence rankings.

### Qualitative analysis

**Figure 1:**
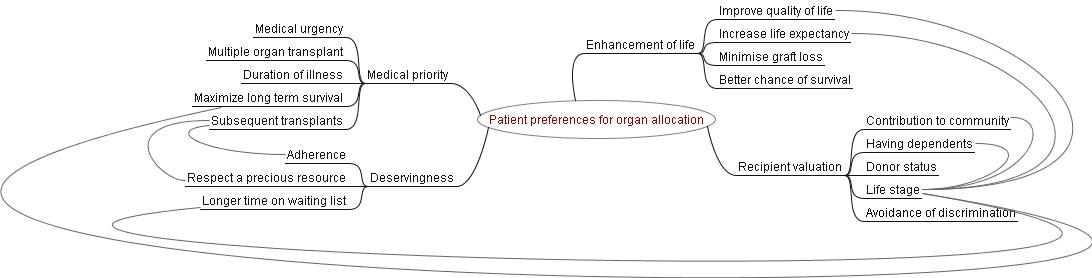
**Thematic schema of themes underpinning patient priority rankings for the allocation of deceased donor kidneys**.

We identified four main themes that underpinned participants ranking of attributes for organ allocation: enhancement of life, medical priority, recipient valuation, and deservingness. These were expressed in the context of the participants' personal experience of illness, profound empathy and desire to gain justice for patients needing a transplant, and their on-going interaction with the health care system. The themes explain the reasoning underpinning the participant's quantitative rankings and largely corroborate the quantitative prioritisation of allocation attributes. The responses from dialysis patients and kidney transplant recipients were broadly similar. Any discrepant views between the two groups are specifically indicated in the text. Illustrative quotations for each theme are provided (See Additional file 1). A thematic schema to illustrate relationships between themes is provided in Figure 1.

### Enhancement of life

#### Improved quality of life

Most participants believed that an organ should be allocated to any person who would gain an improvement in their quality of life with a kidney transplant compared with dialysis; preferentially over a patient whose quality of life would remain the same.

#### Increase life expectancy

The participants felt that a transplant should be given to a person to increase their life expectancy. However, they believed it was inappropriate to deny an organ transplant to one person in favour of another based on an estimated life expectancy as they believed this could not be accurately predicted.

#### Minimise graft loss

Tissue matching was deemed of high importance as a criterion for allocation to minimise the chance of rejection leading to graft loss. This meant reducing the chance that patients would have to endure the trauma of graft loss. The participants indicated that compatibility was an acceptable criterion for prioritising allocation as this could be objectively assessed. They also believed that any illnesses that would affect the new graft should be treated before a patient is allocated a transplant.

#### Better chance of survival

The participants indicated that organs should be allocated to potential recipients who would survive the transplant surgery and have less chance of surgical-related complications. Also, survival meant that the recipient would be able to live a productive and active lifestyle with a kidney transplant. Survival was also related to whether the patient had the capacity to adhere to their medication regimen and clinic appointments after transplantation; either independently or with support from their social networks.

### Medical priority

#### Medical urgency

The participants strongly believed that patients who were medically urgent and unable to survive on dialysis should be given high priority. However, one dialysis patient felt this would be at the expense of patients who were doing well on dialysis as they would have to wait a longer period of time as medically urgent patients would be allocated an organ sooner.

#### Multiple organ transplant

Patients who are waiting for more than one organ transplant (e.g. kidney-pancreas transplant) were deemed "critical" and the participants felt they should receive precedence over those waiting for a single organ. They believed the chances of the transplant being a success would be greater for the recipient of multiple organs from the same donor, who would only undergo one surgery instead of multiple surgeries and be exposed to less risk. The participants believed that if a patient only received one organ, the disease affecting the other organ (e.g. diabetes) may increase a patient's risk of losing their first graft.

#### Duration of illness

The majority of participants stated that length of time since the diagnosis of chronic kidney disease should not be a factor in the allocation process as patients could live normally for a long time before feeling sick and needing dialysis. Rather, time on dialysis was more important as dialysis indicated that the disease was severe and affected a patient's quality of life.

#### Maximize long-term survival

If a transplant candidate had underlying illnesses that would damage the kidney such as obesity or diabetes, the participants believed they should not be prioritised for transplant, particularly if the illness was seen to drastically reduce the patient's chance of long-term survival.

#### Subsequent transplants

The participants felt that the number of previous transplants should not determine priority for allocation. However, they indicated that priority would be given for medically urgent patients but individuals who lost the graft due to non-adherence should receive lower priority in receiving an organ. Many had strong misgivings about organs being allocated to non-adherent or "at-fault" recipients as this was seen to be unfair for compliant patients on the waiting list.

### Recipient valuation

#### Contribution to community

Some participants indicated that the people who could contribute to the community after transplant should receive priority, but acknowledged this would be difficult to judge. However some opposed this view and stated adamantly that allocation should not be determined by a patient's occupation or past.

#### Having dependents

Some participants felt that candidates for transplantation who had family responsibilities (e.g. young children), should be given some priority over those whose did not have dependents. This was not a consensus view. Some believed that it was unjustifiable to discriminate against people who did not have young dependents.

#### Donor status

Some believed that patients should not be given priority based solely on whether they were a registered donor, since some would be medically excluded as organ donors. However, a few mentioned it would be a positive strategy to encourage people to become donors.

#### Life stage

In principle, most participants believed that younger patients should be given priority, as young patients had not experienced life. They knew dialysis impaired growth in children. However, a few older participants on dialysis indicated that in reality, they would be unwilling to give up a kidney to a younger person who had been waiting a shorter time, because older patients would have less chance of being offered a deceased donor kidney again. Some felt that young patients had more opportunities to receive a deceased donor kidney; and that older patients deserved an equal chance of gaining an increased life expectancy with a kidney transplant. Life-stage was an important consideration, and was usually inextricably linked to other concepts such as quality of life, life expectancy, contribution to community, having dependents, and one's personal standpoint. (Figure 1)

#### Avoidance of discrimination

All participants agreed that discrimination based on race, gender or religion was unwarranted and should not be considered in the allocation of organs for transplantation.

### Deservingness

#### Longer time on waiting list

Most participants said that people who have been on the waiting list for the longest period of time deserved to receive some priority in the allocation of organs. Nevertheless, a small minority believed that time on waiting list should not be considered a factor in allocation policy. In their opinion, everyone should have an equal chance of receiving an organ regardless of how long they were waiting and felt that organ allocation should be predominantly based on medical considerations.

#### Adherence

Many were adamant that people who did not comply with their doctor's advice, such as refusing to take their medication or attend dialysis sessions, should not be given priority over those who strictly followed their doctor's recommendations. In particular, transplant recipients emphasised the importance of adherence to post-transplantation medication regimens, and exercise and nutritional recommendations. These participants believed that giving access to transplant, let alone priority, to a "non-adherent" patient would be unfair to other "adherent" patients who were waiting for a transplant. Participants believed that patients had control over their choices, therefore should take responsibility for following medical advice. Participants deemed it "morally wrong" for non-adherent, reckless and self-destructive patients to receive subsequent transplants, particularly if the patient lost the first graft due to a lack of adherence.

#### Respect for a precious resource

Both dialysis and transplant recipients believed that patients on the waiting list who continued to smoke, drink excessively or use illicit drugs should not be waitlisted or receive preference over patients who made "proper" lifestyle choices. Many described how they saw and empathized with, in their view, more "deserving" patients still waiting on dialysis. They felt access to transplantation should be preferentially given to people who were deemed to be living a healthy lifestyle; those who would appreciate and value a precious and scarce resource. Many participants felt that allocating an organ to a patient who did not look after their health was a waste of a valuable resource and was therefore regarded as disrespect to the donor family. However, a few participants admitted they adopted a healthier lifestyle (by giving up smoking, avoiding unhealthy food) only after they had received a transplant.

## Discussion

The majority of patients believed that matching, time on waiting list, medical urgency, likelihood of surviving surgery, age, comorbidities, duration of illness, quality of life, number of organs needed and impact on the recipient's life circumstances were important considerations for the allocation of deceased donor organs for transplantation. Some of these preferences reflect current organ allocation algorithms which are based on time on waiting list, medical urgency, human leukocyte antigen (HLA) matching, sensitization and paediatric status [6]. However, our findings reveal that the patient preferences are defined by their own illness experiences, strong empathy and desire to gain justice for other patients waiting for a transplant, and by their close interaction with the health care system. The rationale for their rankings included: enhancing life, medical priority, recipient valuation, and deservingness. The choices expressed by individuals appeared to be concern for equity (giving a fair chance) over efficiency or for specific sub-groups of patients they believed were justifiably more "deserving" (including patients who had waited longer, adhered to treatment regimens, and valued their transplanted kidney); not as a utilitarian notion of getting the best return possible from the graft.

"Matching" was consistently ranked by patients as the most important criteria for allocation. They perceived this as an objective, straightforward way of allocating organs to minimise poor outcomes. Reducing the chance of rejection leading to graft loss was not rationalised by patients as a means of avoiding the "waste" of a limited resource, rather it expressed as minimising the chance the recipient would endure the trauma of graft rejection. However, it appears that patients may not be aware that with improvements in immunosuppressive agents, the influence of HLA matching on transplant outcomes may be significantly diminished [22].

Broadly, dialysis patients and kidney transplant recipients believed that any individual who would gain life expectancy and quality of life compared with dialysis should be prioritised for transplant. Yet, not all patients who stand to benefit are wait-listed for transplantation. In considering the chances of graft loss as a factor for determining organ allocation, patients anticipated the traumatic impact it would have on the recipients, and rarely expressed this preference in terms of maximising the utility of the graft. Similarly, patient preferences to allocate organs to individuals who were adherent, made healthy lifestyle choices, and had the capacity for self-management post-transplant were strongly driven by the notion to gain fairness for wait-listed patients who would respect and take care of their graft; rather than the need to maximize the utility of a scarce resource. An interesting finding is that transplant recipients ranked first versus subsequent transplants of lower importance, compared to patients on the waiting list, yet, the qualitative data indicated that patients believed the number of previous transplants should not determine priority for transplantation. However, patients often expressed how more "deserving" dialysis patients, i.e. those who would respect a precious resource, missed out when an organ was allocated to a "less deserving" recipient. Patients appeared vehemently opposed to allocating organs to patients who had lost a transplant due to non-adherence or poor lifestyle choices. The difference in priority ranking may reflect that dialysis patients perceive this "injustice" more acutely compared with transplant recipients.

A recent study [3] found that patients were opposed to the use of recipient age as a criteria for organ allocation, including priority for paediatric recipients. Our study reveals that "recipient age" was rarely considered in isolation, but in the context of other criteria for organ allocation including quality of life gains, life expectancy, contribution to community, impact on family, time on waiting list and long-term survival. The patients in our study favoured priority for children as they were aware that dialysis impaired growth. They believed younger people should be prioritised but not at the expense of older aged recipients such that the older person may never have a chance of receiving a transplant. Our findings also reveal an inherent tension where in principle; a patient may agree that priority should be given to younger recipient, but in reality, they would be unwilling to sacrifice their own opportunity for transplant for a younger patient, particularly if the younger patients had been waiting for a shorter time.

This study has some limitations. The participants were English-speaking only therefore the transferability of the study findings to non-English speaking populations is uncertain. Although we aimed to achieve equal proportions of respondents from different patient groups, there were more transplant recipients than dialysis patients. Dialysis patients were sometimes unable to attend due to illness, fatigue and conflicting dialysis sessions. Despite a relatively small number of nominal groups, we reached theoretical saturation, which is when little or no new concepts were arising in subsequent groups. The major strength of this study is the use of a mixed methods (quantitative and qualitative) approach. This approach was useful for corroborating findings, generating more complete data, and attaining enhanced insights regarding the attitudes, beliefs and experiences that underpin patient preferences for allocation criteria. The study involved participants with a range of clinical and demographic characteristics to capture diverse perspectives.

Patient and public preferences for organ allocation [23] share some similarities, for example that social deservingness and recipient valuation should be considered. However, to avoid potential inequity and discrimination, these are not explicitly incorporated in current organ allocation protocols, but may be considered in decisions to wait-list a potential candidate for transplantation. Instead, we would recommend that potential transplant candidates have access to interventions and initiatives to support self-management, adherence and healthy lifestyle choices.

As a key stakeholder group, patients receiving renal replacement should be given the opportunity to provide input in organ allocation policies. As demonstrated in our study, patients can provide relevant, coherent and compelling perspectives in the area of organ allocation. Patient preferences for organ allocation were not based on maximising the utility of a scarce resource, yet this is potentially implicit in current organ allocation systems that aim to balance efficiency and equity. We recommend that organ allocation organisations develop and implement effective strategies for patient involvement. An organ allocation policy that explicitly considers the perspectives, preferences and values of key stakeholder groups, may be deemed more acceptable, transparent and balanced.

Patients believe that an organ should be allocated to a recipient if it confers any quality of life or survival benefit suggesting that organs from marginal donors could be used for transplant [24]. However, to our knowledge, the preferences of potential transplant recipients at the point of care [2], i.e. accepting an offered deceased donor organ, have not been systematically sought. Future research is needed to assess what information patients want in regards to an offered deceased donor organ and how they trade off benefits and risks when deciding to accept organ transplantation. This is particularly important as the donor pool now includes "extended criteria donors" who are older or ill. Compared with recipients who receive "standard" kidneys, extended criteria kidneys are associated with poorer transplant outcomes in terms of patient survival, acute rejection and graft failure; and may place recipients at a higher risk of donor transmitted diseases such as infection or cancer.

## Conclusions

In the professional transplant community, there is an on-going endeavour to strike some reasonable balance between maximising efficiency while achieving equity. It is advocated that policy makers need to be cognisant of patients values and preferences [20]. Some organ allocation organisations may involve patient representatives, however the extent to which patients are involved and their actual contribution is unclear. Dialysis patients and kidney transplant recipients believed that any wait-listed individual who would gain in terms of life expectancy and quality of life compared with dialysis should be prioritised for transplant. Equity of access to transplantation for all patients and justice for those who would look after their transplant were considered important. In contrast, utilitarian rationale based on the maximisation of health gain from the allocation of a scarce resource to avoid "wastage" was rarely expressed. Organ allocation organisations need to seek input from all stakeholders, including patients who can articulate preferences for organ allocation and advocate for equity and justice in the allocation of deceased donor organs for transplantation.

## Competing interests

The authors declare that they have no competing interests.

## Authors' contributions

AT, MI, KH collected and analysed the data. NM conducted data entry. AT/KH drafted the manuscript. All authors critically reviewed and provided intellectual input on the manuscript. All authors read and approved the final manuscript.

## Pre-publication history

The pre-publication history for this paper can be accessed here:

http://www.biomedcentral.com/1471-2369/13/18/prepub

## Supplementary Material

Additional file 1 **Illustrative quotations representing each theme**. Click here for file
