# Supplementary material for: Patient preferences for the allocation of deceased donor kidneys for transplantation: a mixed methods study
Source: BMC Nephrol. 2012 Apr 18;13:18. doi: 10.1186/1471-2369-13-18 (PMC3359260; doi:10.1186/1471-2369-13-18)
Supplement: Additional file 1 — Illustrative quotations representing each theme. [file 1471-2369-13-18-S1.DOC]

**Additional File 1**

**Illustrative quotations representing each theme**

| **Theme** | **Subthemes** | **Illustrative quotation** |
| --- | --- | --- |
| **Enhancement of life** | Improve quality of life | “Even though he has got a shorter time [to live] at least he will have a better quality of life.” (Female, 50s, FG4, dialysis)  “You want somebody to have a lot better life. I mean if someone is really sick then a kidney comes and you’re doing ok and the next person is doing it hard; that person could have a better life.“ (Female, 50s, FG4, dialysis) |
|  | Increase life expectancy | “One could live fifteen years, one could live two years, no one knows how far you’ll come so to say okay, you’re sixty-five you’re going to die soon, you’re thirty you’ve got your whole life ahead of you, that could the sixty-five could live to a hundred and the thirty could get hit by a bus on the way to work so we don’t know” (Female, 40s, FG1, transplant)  “You can’t look at a timeframe [of expected survival] and you can’t answer that question. The doctor’s going to say, ok if you ask me the same thing that’s going to be twenty years with luck. They’re going to look at which [patient] has to gain and how they view it, who should get the kidney.” (Male, 40s, FG1, transplant)  “You cannot say a person’s going to get fifteen years or twenty years, it’s just a hypothetical.” (Male, 40s, FG1, transplant) |
|  | Minimise graft loss | “Definitely compatibility has to be first, you’re not going to transplant a kidney to someone who only has a fifty-fifty chance of it, succeeding.” (Male, 50s, FG4, dialysis) …“You want at least an eighty-five up percentage of it succeeding.” (Male, 50s, FG4, dialysis)  “I think you consider the general health of the person to start with, to work out the complications. I think it’s a close match but also time on waiting list, and I think it goes to who will benefit the most in terms of health.” (Male, 60s, FG3, transplant)  “I think it gets really dangerous when you think outside the science. I hope that the tests are really extensive that they can match it so that this is never an issue where you’re saying oh well you’ve got a family so you must be a better person to have this kidney. I really don’t think that’s very good for anyone else to be making decisions. ‘Best match’ is clear and there’s no trade-offs”. (Female, 20s, FG3, transplant) |
|  | Better chance of survival | “Survival of surgery’s there. If you’re not going to survive surgery, you’re not going to do anything. So I’d shift that up [the priority ranking].” (Male, 50s, FG2, transplant)  “They’ve got to be mentally sufficient to handle it and that they will they be able to take the drugs if they live by themselves or that sort of thing. And will take the drugs.” (Male, 50s, FG2, transplant)  “I wrote down health and then I prefaced that with potential of long term survival of the patient.” (Male, 50s, FG5, dialysis) |
| **Medical Priority** | Medical urgency | “I don’t think I could choose but I think the person that’s the sickest, I would tend to lean towards.” (Female, 40s, FG1, transplant)  “Basically my doctor said to me, ‘Oh you’re the healthiest patient I’ve got.’ I said, ‘well what does that mean?’ At the end I was like what he said, it’s not good. Because, that means you get put behind because other patients are sicker than you and I thought ok.” (Female, 40s, FG4, dialysis)  “The doctor says, ‘you’re the second really good healthy patient that I’ve seen today.’ I just thought is that good or bad? He said, ‘what do you mean? It means you’re the best.’ I said, ‘yeah that’s terrific but it means I’m really healthy so I’m staying on this list for a long time while you look after everybody else.’ And, he just said, ‘um, no comment.’” (Male, 50s, FG4, dialysis) |
|  | Multiple organ transplant | “Well it you have a situation where a donor’s got two kidneys and a pancreas, well you could give that pancreas and one kidney to that one recipient straight away. People use the best of the organs, of all of the organs available.” (Male, 50s, FG1, transplant) |
|  | Duration of illness | “You can live with illness for a long time before they actually put you on the waiting list.” (Male, 40s, FG3, transplant)…”Yeah, I had mine twenty-two years and when I was, for the kidney, my time on the waiting list was fifteen years certainly the same! So time on waiting list was a lot harder than the duration of the illness for the previous twenty-two.” (Male, 60s, FG3, transplant) |
|  | Maximize long-term survival | “They could have diabetes, they could have heart problems, they could have a lot of other problems and given maybe another twelve months to live. What are you going to do then? That’s why they can’t get priority. Because that’s what we coming here for you give them a kidney and they’re going to die anyway in the next twelve to eighteen months.” (Male, 60s, FG3, transplant) |
|  | Subsequent transplants | “Yeah, I don’t think it’s the number [of transplants they’ve had] it’s whether they looked after it or not.” (Female, 40s, FG3, transplant)  “Think we’ve all seen that happen. I’ve noticed people that have a kidney transplant and they haven’t done the right thing and they’ve lost it and they’re back in rehab and looking for a second kidney transplant. I said why didn’t you look after the first one?” (Male, 60s, FG5, dialysis)  “I knew two other people on dialysis up here at the hospital. One of them had his third kidney transplant and one of them had his second kidney transplant and both are back on dialysis and the reason why they were is because they didn’t look after themselves. Now, for them to get another kidney, I think it would be morally wrong, but you know, because they weren’t looking after themselves. If you look after yourself, if you take your immunosuppressants all the time, if you exercise, if you do all these other things, there’s no logical reasons why your kidney shouldn’t last you for the rest of your life.” (Male, 60s, FG3, transplant) |
| **Recipient valuation** | Contribution to community | “A thirty-year-old would be working [should get priority], yeah, he’d be more productive. Compared to a sixty-five, he’d be retired. Hopefully.” (Male, 50s, FG1, transplant)  “Well, you wind down, you start to kick back and it becomes more very centred around your immediate family and you don’t really go out and push yourself out into the community as much.” (Male1, 50s, FG4, dialysis) …”But, for some people when they retire, they do, do a lot of community work.“ (Male, 60s, FG4, dialysis)  “If you’ve got somebody that’s been breaking the law then there should be no advantage over the person who’s upholding the law and being of benefit to the community.” (Male, 50s, FG5, dialysis) |
|  | Having dependents | “there is an issue where you’re saying, ‘oh well you’ve got a family so you must be a better person to have this kidney.’ I really don’t think that’s very good for anyone else to be making decisions.” (Female, 20s, FG3, transplant)  “Well you’d be going with the one that’s twenty-five because they are more likely to have younger kids, if you’re sixty or sixty five your kids are already in their twenties.” (Male, 60s, FG4, dialysis)” |
|  | Donor status | “Well in that case it all depends if they can donate because some people can’t.” (Male, 50s, FG4, transplant) |
|  | Life stage | “I certainly think children should [receive priority] because their growth is affected.” (Female, 50s, FG5, dialysis)…”I’d go along with that too.”(Male, 60s, FG5, dialysis)…“So, if you knew you were in line to get a kidney and they said to you, ‘you can have one or we’ve got this 12 year old who also needs it but he’s only just gone on the list,’ you’d be willing to say ‘oh, he can have it,’?” (Male, 50s, FG5, dialysis) replies …”No, I would never say that, I’d take the kidney.” (Male, 60s, FG5, dialysis)  “If you have two people and an old one and a young one who match equally, I think that the young one gets it. And I don’t object to it but I think this is what happens.” (Female, 70s, FG3, transplant)  “I’m an old person but I think that if a young person needed a cadaver, a younger, quite a bit younger like a forty-year-old or thirty-year-old and I was ahead of them and they needed an organ, I think they should take priority over the old one. I don’t like the idea.” (Female, 70s, FG3, transplant)  “The opposite argument also prevails is that if you’ve got a five-year-old person and a seventy-year-old person, the five-year-old person’s got much longer time to get a kidney transplant but a seventy-year-old not necessarily” (Male,60s, FG3, transplant)  “As long as the old person isn’t going to get bumped off the list as a result because they are now too old.” (Male, 50s, FG4, dialysis)  “No if you’re healthy and you can pass the rigours of the walking the treadmill and so forth to make you are staying fit, which I’ve got to do, and I don’t feel my age, so if I am waiting and this kidney turns up for me, I’ll take it. It doesn’t matter that I’m 67 or whatever.” (Male, 50s, FG5, dialysis)  “I was never certain about the waiting list but, I thought that a young good kidney should go to a young healthy person. Probably because I was in my sixties, I didn’t mind if I got somebody’s pickled kidneys as long as it could get me going for a few years.” (Male, 60s, FG1, transplant)  “I think you know, if that means that the old person has got to wait for the next one that matches, which might be another six months or whatever, if they are that close up the list, yeah that’s ok. But if that means that is going to push them over the limit, so they are now too old and not going to give them one at all then I’d say in that situation they should get [the transplant]” (Male, 50s, FG4, dialysis)  “I'd say at the moment yes [give it to the younger patient]. But I've only been waiting 12 months. That person's got a lot longer life ahead of them hopefully than what I have. So at the moment I would say yes give it to that younger person. Because, as long as I'm still at the top of the list, the next one available may suit me.” (Female, 60s, FG6, dialysis)  “Let the younger ones have a life.” (Female, 60s, FG4, dialysis) |
|  | Avoidance of discrimination | “That [gender] shouldn’t be part of the equation, no.” (Male, 50s, FG2, transplant)  “Well that’s, well race and religion shouldn’t really come into it at all” (Male, 50s, FG4, dialysis) |
| **Deservingness** | Longer time on waiting list | “Yeah, definitely, definitely. The length of time on the list should come into it, at some stage” (Male, 50s, FG4, dialysis)  “[Medical urgency is more important] I don't think time on the waiting list is so important. You're on it. (Female, 60s, FG6, dialysis) |
|  | Adherence | “I thought to receive one, if you were more likely to follow the doctor’s instructions and try to take your meds when you’re supposed to, if they say cut out meat and you do all the things. Like I always said if they told me to stand on my hands I would do that if it got me further, or a better chance of having a transplant.” (Female, 40s, FG1, transplant)  “When I had my transplant, it was five days afterwards, there was a young guy who had polycystic kidney disease. He was about twenty-two, he was a surfer, he was doing haemo[dialysis], he didn’t always turn up at clinic to have his haemo[dialysis], he was drinking Red Bull, um, eating Vegemite, he ate a full box of chocolates on time and made himself sick in the ward. He wouldn’t do anything unless he wanted to do it and he was whinging why can’t I get a kidney and these sorts of things but he was doing everything to counter what everyone was trying to tell him to do for his benefit. And, you know, I could see that if they had the offer to give to someone that was going to follow instructions and one that was not, that they would give it to the one that was more likely to do everything to keep the kidney than he was. It was like he was self-destructing all the time and he was only young.” (Female, 40s, FG1, transplant)  “I had two other people on dialysis up here at the hospital. One of them had his third kidney transplant and one of them had his second kidney transplant and both are back on dialysis and the reason why they were is because they didn’t look after themselves. Now, for them to get another kidney, I think it would be morally wrong, but you know, because they weren’t looking after themselves. If you look after yourself, if you take your drug, immunosuppressants all the time, if you exercise, if you do all these other things, there’s no logical reasons why your kidney shouldn’t last you for the rest of your life.” (Male, 60s, FG3, transplant)  “Yeah, one of my nurses told me [not following medical advice] was one of the number one causes for rejection. And it was the saddest reason of them all because it was something you could physically do and control.” (Female, 40s, FG1, transplant) |
|  | Respect a precious resource | “I think those who, like do the right thing, should be in consideration along before those who you know, continually drink and smoke and that, they shouldn’t and they do it” (Female, 50s, FG4, dialysis)  “It depends on their lifestyle. If he's an alcoholic or a smoker or a drinker, no. I've looked after myself for this all my life. But if he's a young, healthy and this [kidney disease] has happened to him. Well, it's happened to my boys. They've got polycystic kidneys and they're going to go in for kidney failure. No fault of their own. I'd give it to them.” (Female, 40s, FG6, dialysis)  “Even if they get a kidney and they start smoking or they start the drugs again, that could’ve gone to someone who would’ve appreciated it to the fullest and it’s just a waste of a kidney and a waste of a family’s thinking that their son or daughter has died knowing that something good has come across from it. I think you have to be really prepared for a transplant and give it all you’ve got to make it last. You can’t get it and oh, and fine I can start smoking, I can start drugs, I don’t need my medication, I’m you know, medication’s just you know, I’ll go see the doctor. You’ve just got to be aware.” (Female, 40s, FG1, transplant)  “Yeah, when I’d done my transplant, this guy got it the same time as me and he went down the lift and he went for a walk and he went where they had the cigarettes and that and he went there and had a cigarette.” (Male, 50s, FG1) …”Oh, that would have been horrible.” (Female, 60s, FG1, transplant)  “As you said if a person doesn’t look after themselves well you’re tempted to say well no sorry.” (Male, 50s, FG4, dialysis) |
